# Analysis and design of holographic magnetic metasurfaces in the very near field for sensing applications at quasi-static regime

**DOI:** 10.1038/s41598-023-36452-2

**Published:** 2023-06-07

**Authors:** Martina Falchi, Sabrina Rotundo, Danilo Brizi, Agostino Monorchio

**Affiliations:** 1 Department of Information Engineering, University of Pisa, 56122 Pisa, Italy; 2 Consorzio Nazionale Interuniversitario per le Telecomunicazioni (CNIT), 43124 Parma, Italy

**Keywords:** Engineering, Materials science

## Abstract

In this paper, we present a novel low-frequency sensing solution based on the manipulation of the near-field distribution by employing a passive holographic magnetic metasurface, excited by an active RF coil placed in its reactive region. In particular, the sensing capability is based on the interaction between the magnetic field distribution produced by the radiating system and the magneto-dielectric inhomogeneities eventually present within the material under test. We first start from conceiving the geometrical set-up of the metasurface and its driving RF coil, adopting a low operative frequency (specifically 3 MHz) to consider a quasi-static regime and able to increase the penetration depth within the sample. Afterwards, since the sensing spatial resolution and performance can be modulated by controlling the metasurface properties, the required holographic magnetic field mask, describing the ideal distribution at a specific plane, is designed. Then, the amplitude and phase of currents, flowing in each metasurface unit-cell and required to synthetize the field mask, are determined through an optimization technique. Next, the capacitive loads necessary to accomplish the planned behavior are retrieved, by exploiting the metasurface impedance matrix. Finally, experimental measurements conducted on fabricated prototypes validated the numerical results, confirming the efficacy of the proposed approach to detect inhomogeneities in a medium with a magnetic inclusion in a non-destructive manner. The findings show that holographic magnetic metasurfaces operating in the quasi-static regime can be successfully employed for non-destructive sensing, both in industrial and biomedical fields, despite the extremely low frequencies.

## Introduction

Electromagnetic sensing is a dramatically raising research field in the scientific community, due to the countless applications in which it is involved. Not only biomedical, but also industrial environments are significantly pushing the research activity in this sense. In particular, developing techniques and tools to perform inspections and characterizations of materials and structures without impairing their usefulness or causing irreversible damage is fundamental for industrial non-destructive testing and evaluation (NDT&E)^1–3^. Indeed, one of the manufacturing industry’s primary goals is to ensure product quality, or the ability of the product to perform its intended function for an extended period of time. Still nowadays, visual inspection by production or maintenance personnel is the most often utilized NDT approach ^4^, but it is frequently combined with other techniques due to its intrinsic limits. At the same time, the operative sensing principles underlying various NDT methods are widely applied in multiple technological fields, not only in manufacturing. As mentioned above, biomedical imaging is also a very important field of application; as a matter of fact, tumors, fractures and other abnormalities are diagnosed using X-rays or magnetic fields^5–13^.

Therefore, the overall procedures that account for the majority of the market are radiography^14–16^, ultrasonics^17,18^, eddy current^19–21^, magnetic particles^22–24^ and penetrant testing^25,26^. In this broad context, electromagnetic imaging is a technique for performing non-destructive testing (NDT) and biomedical imaging by observing the material’s electromagnetic response. It retains several advantages compared against other techniques, especially in terms of costs, instrumentation simplicity, accuracy and safety with respect to human body interactions.

More specifically, the electromagnetic response can be expressed as changes in the radiating system complex reflection coefficient, due to impedance amplitude or resonant frequency variations. Such phenomenon is related to the interactions of the produced electromagnetic field with the material under test (MUT). Several solutions have been proposed in the literature so far, and the electromagnetic frequencies that can be used for sensing applications nearly cover the whole electromagnetic spectrum^27^. Namely, the open-ended rectangular waveguide is a very common technique in NDT due to its simple structure, ease of fabrication, and low costs^28^. The resolution of the rectangular waveguide probes is comparable to the larger dimension of the waveguide aperture. As a result, a large operative frequency is required to achieve a sufficiently high resolution. On the other hand, high frequencies reduce signal penetration depth within the MUT, thereby limiting the probe application range. Additionally, as the frequency increases, the costs and complexity of the system implementation are raising as well.

Besides, a wide range of electromagnetic sensors has been also developed, such as single or linear arrays of resonating unit-cells. Among these cells, square or circular spiral resonators are the most common solutions. This kind of probes provide a high miniaturization rate compared with other resonant and non-resonant structure; moreover, spiral resonators probes can be excited through a single port, thus reducing the complexity and the costs of the system. Nonetheless, technological methods relying on a single resonating sensor^29^ or a linear array of resonating sensors^30^ have limited reconfigurability and do not allow for rapid scanning of large areas^31^.

To overcome the aforementioned limitations, we propose a near-field passive holographic magnetic metasurface for electromagnetic sensing based on the low frequency field distribution manipulation, suitable for both non-destructive testing and biomedical imaging applications. In recent decades, metamaterials and metasurfaces have become a popular topic in the scientific community due to the flexibility they provide in manipulating the entire electromagnetic spectrum, a property not possessed by conventional materials^32^. According to the most recent research studies, these structures, both in their three-dimensional and two-dimensional forms, can be engineered to create innovative sensing devices^33–36^ or to give life to novel calculation tools^37^. Magnetic metasurfaces, in particular, have been explored for their intriguing features in relation to the impinging magnetic field^38–41^. As a result, magnetic metasurfaces enable the manipulation of magnetic fields with a degree of flexibility that is not achievable with ordinary materials. As illustrated in^42^, it is possible to control and modify the field distribution of magnetic metasurfaces in the low-frequency region, i.e. at quasi-static regime. In^43,44^, holographic metasurfaces are designed to manipulate the field around an assigned volume at 20 GHz; herein, the operating frequency is set to 3 MHz, so the size of the overall radiating system (unit-cells of the metasurface and RF exciting coil) is much smaller than λ, and we primarily work with the magnetic near-field components. Thus, a metasurface, capable of performing sensing with sufficient spatial resolution while maintaining low complexity and costs, is herein proposed and designed. We demonstrate how to determine the optimal current choices, in terms of amplitude and phase, for each unit-cell element in order to produce a hologram over the metasurface by spatially filtering the magnetic field produced by the actively fed RF coil. The desired magnetic field distribution is obtained by using an analytical formulation based on a magneto-static approach. To fully describe the metasurface capabilities, we propose two extreme configurations. The first one generates an ultra-focused magnetic field distribution, allowing for fine sensing of small and localized areas. The second one generates a homogeneous magnetic field distribution, which enables the full metasurface area to be used for sensing applications.

The paper is organized as follows. Firstly, a detailed statement of the problem with the specific aim of the work is reported. Then, the analytical framework for the control of the magnetic metasurface and the synthesis of the desired magnetic field distribution via the Biot-Savart formulation is developed. As the following, the numerical model design and prototype fabrication procedures are described. A section is specifically dedicated to the discussion and comparison of numerical and experimental results. Finally, conclusions are derived.

## Results and methods

### Statement of the problem

**Figure 1:**
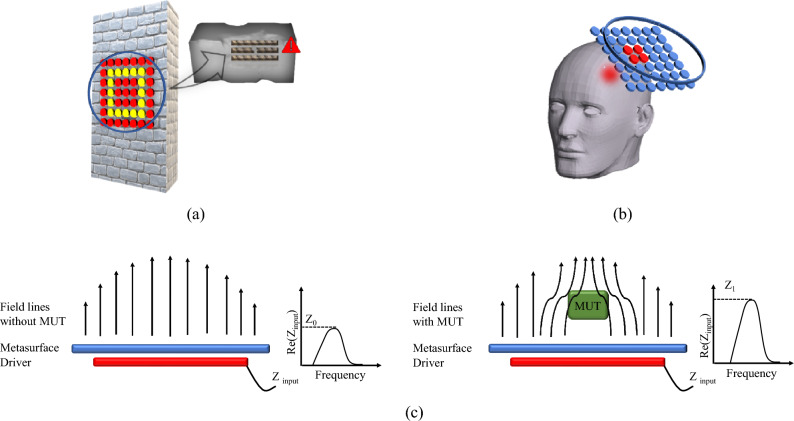
Pictorial representation of two possible applications of the metasurface-based proposed system: fracture identification in reinforced concrete structures (**a**) and brain tumor detection (**b**); schematic of the working principle of the radiating system (**c**).

As reported in the Introduction, the purpose of this study is to develop a passive magnetic metasurface for electromagnetic sensing in the quasi-static regime able to manipulate the spatial distribution of its near-field. Specifically, the metasurface spatially filters the magnetic field produced by a closely placed active RF coil^45^. As shown in Fig. 1, where a conceptual drawing of the proposed system is depicted, the near-field manipulating magnetic metasurface is meant to be used for both industrial non-destructive testing (for instance, fracture identification in reinforced concrete structures) and biological imaging applications (as in brain tumor detection). In both applications, the control parameter is the variation of the input impedance of the RF exciting coil; as can be seen from Fig. 1c, the presence of the MUT deforms the field lines produced by the radiating system and consequently the output signal from the driving coil.

As it is well known, with a progressively lower operating frequency, field manipulation becomes a more difficult task. Indeed, the corresponding wavelength is, in turn, progressively larger, and no phase compensation, as traditionally followed in array theory, can be exploited^46–48^. In this manuscript, the wavelength at the operating frequency range (around 3 MHz) is 100 m. Despite this, we herein design a magnetic metasurface able to spatially filter the magnetic field distribution produced by a closely placed active RF coil in an arbitrarily way (as required for the specific use case). This implies that, differently from the works appeared so far in the literature about arrays, a near-field manipulating capability can be achieved even if the radiating system dimensions are strongly in the subwavelength regime, with an extremely reduced spatial resolution-wavelength ratio. In other words, a magneto-static approach is employed and the focusing capabilities are achieved without recurring to the traditional phase compensation adopted in array theory.

Moreover, the herein presented approach also enables the determination of the reactive loads required to generate the desired magnetic field distribution (that can be in the form of a localized spot or, on the contrary, a homogeneous field) at the target geometrical plane. Hence, a complete design flowchart can be accomplished, from the desired metasurface behavior conception to its actual implementation.

### Analytical formulation

Without losing generality, we assume that the metasurface is made by a matrix of unit-cells, as for instance resonating spiral or split ring resonators. Moreover, we can also assume that a magneto-static approximation can be applied, provided that the dimensions of the overall radiating system are maintained significantly smaller with respect to the operating wavelength (3 MHz). Therefore, once the geometrical configuration of the radiating system has been set, it is possible to analytically implement the Biot-Savart formulation in a coding environment, as reported in the following equation.

1 $$\vec{B}\left( {\vec{r}} \right) = \frac{{\mu_{0} }}{4\pi }I\oint {\frac{{d\vec{l} \times \overrightarrow {{r^{\prime}}} }}{{\left| {\overrightarrow {{r^{\prime}}} } \right|^{3} }}}$$

where ${\mu }_{0}$ is the vacuum magnetic permeability, *I* a filamentary current and ${r}^{^{\prime}}$ the distance between the infinitesimal current path element $d\overrightarrow{l}$ and the spatial point in which the resultant magnetic field *B* is computed. The total magnetic field distribution at a particular geometrical plane can be determined by superimposing the Biot-Savart evaluation for each element constituting the overall radiating system and for a unit circulating current. In this way, the total magnetic field distribution can be weighted by opportunely selecting the currents flowing through the radiating system’s constituents.

**Figure 2:**
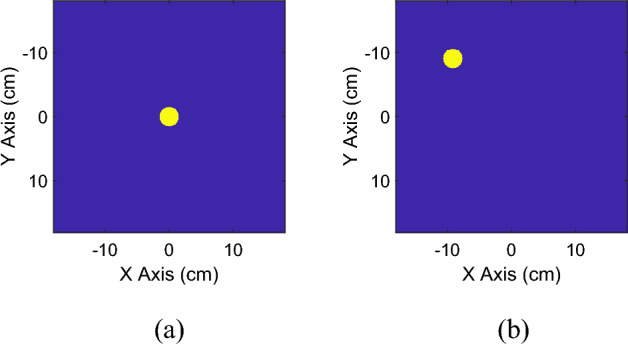
Example of binary masks with a central spot (**a**) and with a spot in the upper left corner (**b**). The masks represent the desired magnetic field distribution at the target plane ($z=4 \mathrm{cm}$).

Our aim is to produce a hologram over the metasurface by employing binary masks to create the desired magnetic field distribution (as shown for instance in Fig. 2) at a certain target plane (herein chosen at $z=4 \mathrm{cm}$). These matrices, which have the same geometrical dimensions as the field distributions calculated through Biot-Savart formulation on the target plane, are equal to zero everywhere except for the region where the magnetic field presence is desired (equal to 1). By referring to Fig. 2, the desired focal spot has a 3 cm diameter. As a result, an optimization algorithm technique can be employed to determine the amplitude and phase of the currents at each element of the system capable of synthesizing the desired magnetic field distribution at the target plane. A square root minimization algorithm can be employed to reduce the distance between the synthetized field and the mask.

Finally, a full-wave simulation is performed to retrieve the impedance matrix of the unloaded metasurface and the exciting RF coil. To determine the reactive loads to be set in the numerical model to recreate the optimized currents distribution (and, consequently, the desired magnetic field), it is necessary to recall the Kirchhoff relations, expressed in matrix form.

2 $$\left( {\begin{array}{*{20}c} {Z_{11} } & {\quad Z_{12} } & {\quad Z_{13} } & {\quad \ldots } & {\quad Z_{{1\left( {N + 1} \right)}} } \\ {Z_{21} } & {\quad Z_{22} } & {\quad Z_{23} } & {\quad \ldots } & {\quad Z_{{2\left( {N + 1} \right)}} } \\ {Z_{31} } & {\quad Z_{32} } & {\quad Z_{33} } & {\quad \ldots } & {\quad Z_{{3\left( {N + 1} \right)}} } \\ \vdots & {\quad \vdots } & {\quad \vdots } & {\quad \vdots } & {\quad \vdots } \\ {Z_{{\left( {N + 1} \right)1}} } & {\quad Z_{{\left( {N + 1} \right)2}} } & {\quad Z_{{\left( {N + 1} \right)3}} } & {\quad \ldots } & {\quad Z_{{\left( {N + 1} \right)\left( {N + 1} \right)}} } \\ \end{array} } \right)\left( {\begin{array}{*{20}c} {I_{1} } \\ {c_{2} I_{x} } \\ {c_{2} I_{x} } \\ \vdots \\ {c_{{\left( {N + 1} \right)}} I_{x} } \\ \end{array} } \right) = \left( {\begin{array}{*{20}c} {V_{1} } \\ 0 \\ 0 \\ \vdots \\ 0 \\ \end{array} } \right)$$

In (2), index 1 refers to the exciting RF coil and *N* indicates the number of unit cells of the metasurface; the system’s applied voltage is known, as it refers to the driver’s voltage source. In particular, we indicate the currents flowing in the metasurface unit-cells through a linear combination based on the current coefficients *c*_*i*_^49^, that are the parameters to be determined by the optimization algorithm. Since the impedance matrix for the unloaded configuration is known from the full-wave simulation, the capacitive loads required for accomplishing the designed behavior for each metasurface unit-cell can be easily retrieved, as described below.

3 $$Z_{ii} = \frac{{ - \mathop \sum \nolimits_{j = 2,j \ne i}^{N + 1} c_{j} Z_{ij} }}{{c_{i} }}\quad with \quad i = 2,3 \ldots \left( {N + 1} \right)$$

Clearly, at a first approximation, resistive losses can be neglected, since they are typically far smaller than the reactive impedances values.

### Design procedure

#### Numerical design

**Figure 3:**
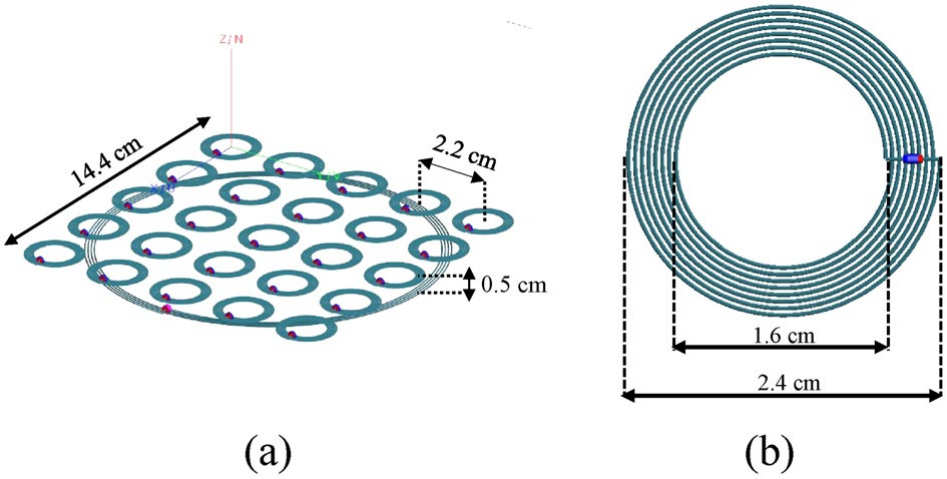
3D CAD model of the overall radiating system: the proposed 5 × 5 passive metasurface is placed above the actively fed RF coil (**a**); metasurface 8-turn unit cell detail (**b**).

To demonstrate the validity of the suggested approach, we developed a numerical test-case by exploiting a Method of Moments full-wave simulator (Feko suite, Altair, Troy, MI, USA). We choose a low operative frequency (3 MHz) to maximize the penetration depth inside the material under test (MUT); as discussed, the spatial resolution is ensured by the field distribution control, despite the relatively long wavelength. All the radiating system components, shown in Fig. 3a, were designed by adopting a single strand 28 AWG lossy copper wire with a radius of 0.16 mm. In particular, the active RF coil was constructed as a 4-turn planar spiral with an external diameter of 14.4 cm and an internal diameter of 13.4 cm. Theoretically, to develop a metasurface for sensing purposes, an infinite array of unit cells would be required to properly satisfy the ideal hypothesis mentioned in^42^ and prevent undesired truncation effects. Clearly, such design is not practical, and there are limitations to consider. As a matter of fact, to prevent significant ohmic losses and allow its integration in real-scenario applications, the metasurface must be as compact as possible with a limited number of unit cells. More importantly, it is worth to underline that the metasurface is excited by the driver’s near-field, which is different from a plane wave excitation.

As a result, we realized a 5 × 5 holographic metasurface with an overall external dimension of 14.5 cm × 14.5 cm. As illustrated in Fig. 3b, each unit cell was made of an 8-turn spiral resonator with an external diameter of 24.16 mm and a 520 µm pitch, separated by the adjacent element by a 6 mm gap. The metasurface was placed 5 mm above the exciting RF coil. Table 1 summarizes the system design parameters.

**Table 1:** System design parameters adopting single strand copper wire.

Parameters	Driving coil	Unit cell
Outer diameter (cm)	14.4	2.4
Inner diameter (cm)	13.4	1.6
Number of turns	4	8
Copper wire	28 AWG	28 AWG
Source impedance (Ω)	50	N/A
Working frequency (MHz)	3	3

As mentioned in the Introduction, we arranged two different extreme radiating configurations case studies. The former generates a focused field distribution, and it is suitable to perform a spatial localization of the inhomogeneities within a MUT. The corresponding field mask has been reported in Fig. 2b. Conversely, in the latter arrangement the field distribution is homogeneous, i.e., with all the metasurface unit-cells presenting the same circulating current, thus allowing the entire metasurface area to be used for sensing. In the following section, these two configurations will be presented and discussed, highlighting the corresponding advantages and disadvantages.

#### Prototype fabrication

**Figure. 4:**
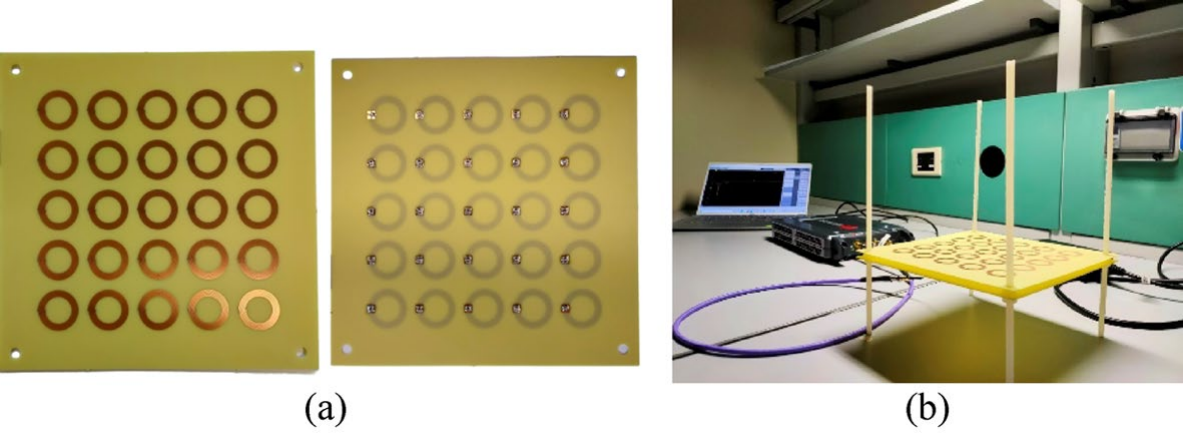
Experimental set-up: (**a**) picture of the PCB metasurface slab (front and back views); (**b**) measurement set-up of the complete radiating system (driving coil and metasurface).

To create an accurate, repeatable, and mechanically robust prototype, all system components (driver and metasurface) were manufactured by using PCB technology, although the corresponding ohmic losses are higher with respect to employing single strand copper wires^13,50,51^. The chosen dielectric substrate was FR4 (a composite material made by a glass fiber fabric impregnated with a flame-retardant epoxy resin matrix, ${\varepsilon }_{r}=4.2$) with a thickness of 0.8 mm, above which 35 µm thick copper strips were etched (see Fig. 4a). Table 2 summarizes the experimental system design parameters.

**Table 2:** System design parameters for PCB manufacturing.

Parameters	Driving coil	Unit cell
Outer diameter (cm)	14.4	2.4
Inner diameter (cm)	13.4	1.6
Number of turns	4	8
Metal line width (mm)	0.32	0.32
Spacing between windings (mm)	0.93	0.2
Conductor thickness (µm)	35	35
Source impedance (Ω)	50	N/A
Working frequency (MHz)	3	3

Since the strip line configuration leads to a slightly different inductive behavior of the system components, it was required to evaluate again the capacitor values for both the above mentioned metasurface setups (i.e., “homogenized” and “focused”).

Finally, we soldered the retrieved surface-mount capacitors over the boards, and we connected the driving coil to a VNA (VNA P9374A, 300 kHz–20 GHz, Keysight, USA) by using a 50 Ω micro SMA connector (Fig. 4). In order to guarantee driver and metasurface axiality and relative distance, each substrate slab was provided with four external holes to create a nylon support (Fig. 4).

## Results

### Numerical results

**Figure 5:**
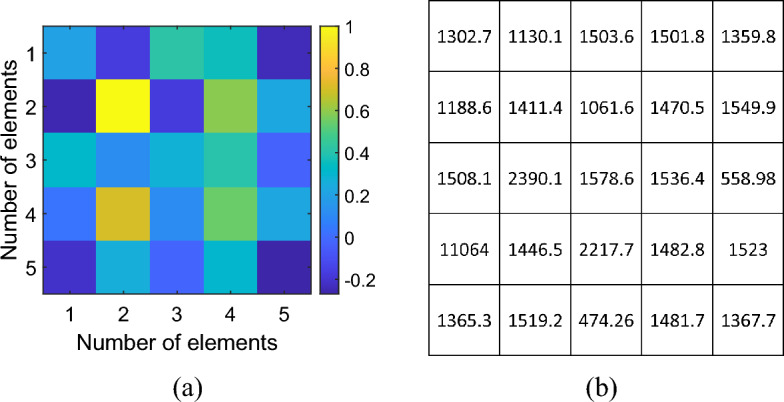
(**a**) Normalized currents map calculated through the proposed optimization algorithm. The map allows to evaluate the spatial correspondence between each cell of the metasurface and the current value required to obtain the desired focused magnetic field distribution. (**b**) Capacitance values (pF) used to load the metasurface unit-cells in order to satisfy the optimized current values and to focus the magnetic field distribution.

**Figure 6:**
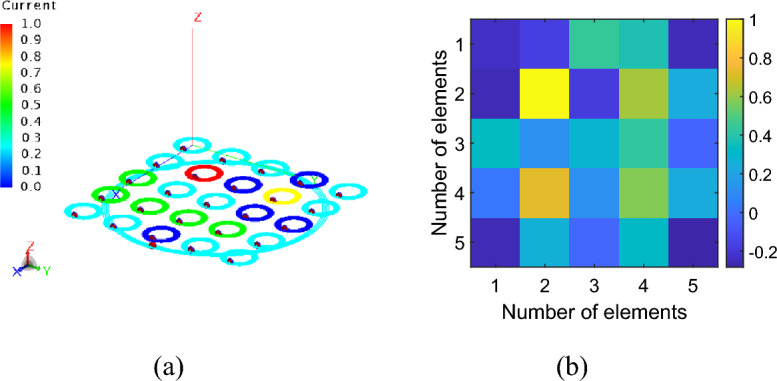
Current distribution within the metasurface elements obtained from a full-wave simulation (**a**) and corresponding currents map in amplitude and phase (**b**).

**Figure 7:**
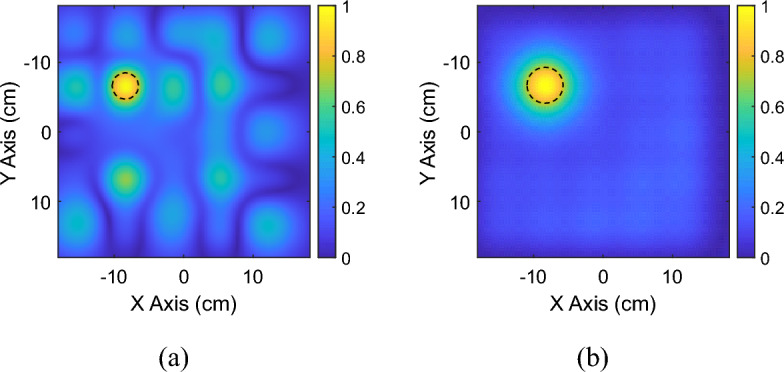
Focused magnetic field distribution (magnitude) at a plane 4 cm distant from the metasurface obtained with the optimization algorithm procedure (**a**), and by activating only one unit-cell of the metasurface (**b**).

As described in the previous section, the capacitive loads required to shape the magnetic field distribution have been calculated starting from Eq. (3), considering the current values determined by the optimization algorithm. In particular, the currents and the corresponding capacitance values retrieved for the magnetic field mask depicted in Fig. 2b are shown in Fig. 5a,b, respectively. To achieve the desired focused magnetic field distribution, the driver was also involved in the currents optimization process. For this reason, the RF exciting coil was loaded with an 827 pF capacitor in series with a 0.15 Ω resistor which simulates the lumped element loss factor. Then, we performed a full-wave simulation at the working frequency of 3 MHz, evaluating both the currents distribution in the unit-cells and the obtained magnetic field maps. In particular, the currents distribution retrieved with the optimization analytical model (MATLAB, Fig. 5a) and with the full-wave simulations (Feko, Fig. 6b) revealed an excellent correlation coefficient, greater than 80%. In the same simulation, it was also determined whether the magnetic field distribution generated by the system matched the desired one at the target plane at 4 cm, depicted in Fig. 2b. By observing the full wave obtained magnetic field distribution in Fig. 7a, it is possible to notice that, at the desired plane, satisfactory matching with the binary mask is achieved. Additionally, we also report a comparison against a simplified scenario where no optimization procedure is adopted and only a single unit-cell is activated at the desired focal point, being the other unit cells, instead, kept off resonance (Fig. 7b). As apparent, by following the currents optimization approach presented in this work, we are able to create a hotspot 54% smaller than the scenario where only one unit-cell was activated, matching very closely the desired mask (Fig. 2b). Therefore, the procedure is effective in significantly improving the system sensing spatial resolution. It is worth to point out that such focusing capability has been achieved at 3 MHz, where the $1/{r}^{3}$ near-field components dominate. In this sense, considering that the metasurface unit-cells can be approximated by magnetic dipoles, we exploited the dependence of the magnetic field ${H}_{r}^{\left(m\right)}$ on the $cos\left(\theta \right)$ component, according to the following formula.

4 $$H_{r}^{\left( m \right)} = \frac{1}{{\zeta_{0} }}\frac{{I_{m} \Delta z}}{2\pi }\left( {\frac{1}{{r^{2} }} + \frac{1}{{jk_{0} r^{3} }}} \right)\cos \theta e^{{ - jk_{0} r}}$$

where with $\theta$ we refer to the inclination of the dipole with respect to its main axis, ${\zeta }_{0}$ is the characteristic impedance of free space, ${I}_{m}\Delta z=j\omega \mu SI$ is the magnetic moment of the dipole, with $\mu$ magnetic permeability of the surrounding medium and $S=\pi {R}^{2}$ section of the elementary coil, ${k}_{0}$ is the wave number in free space and *r* is the radial distance from the dipole. Thus, the choice to position the metasurface unit-cells on the *xy* plane (Fig. 6a), allow us to obtain the maximum radiation of the magnetic near field. This field configuration can be useful for an extremely targeted sensing procedure; in particular, by dynamically reconfigure the focal spot, a progressive and accurate scan of a MUT may be also pursued.

**Figure 8:**
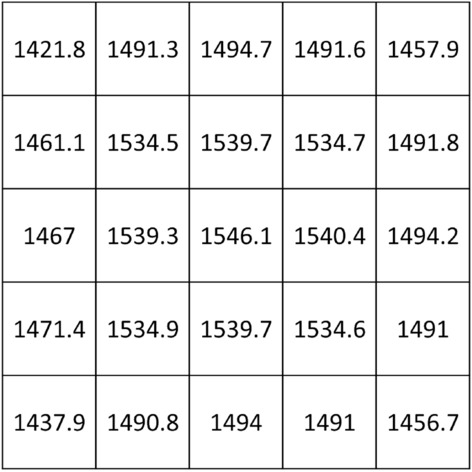
Capacitance numerical values (pF) used to homogenize the near field distribution of the metasurface.

**Figure 9:**
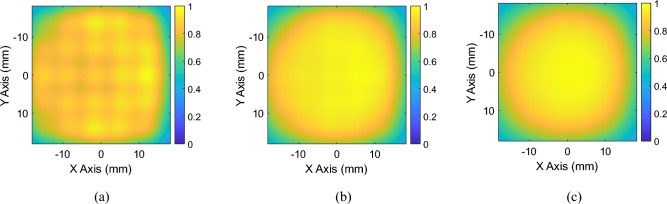
Magnetic field distribution at a plane 4 cm (**a**), 5 cm (**b**) and 6 cm (**c**) away from the metasurface for the homogeneous configuration.

As stated in the section devoted to the analytical formulation, the other tested configuration consists in a metasurface producing a homogeneous near field. To achieve this goal, the currents flowing in each element of the metasurface must be equal each other. Therefore, by following the previously described theoretical model, each cell of the metasurface was loaded with the proper capacitance value showed in Fig. 8. By evaluating the obtained magnetic field maps (Fig. 9), a satisfying homogeneous distribution can be observed, progressively worsening with the distance from the metasurface plane. In this case, where the magnetic field distribution is extended over a larger area, the entire dimension of the metasurface can be utilized for sensing applications, thus increasing the geometrical portion of a MUT that can be analyzed.

### Experimental results

The two systems to be tested are connected to the VNA, coupled through a connector soldered in the back of the driver for both the prototypes, as previously described.

**Figure10:**
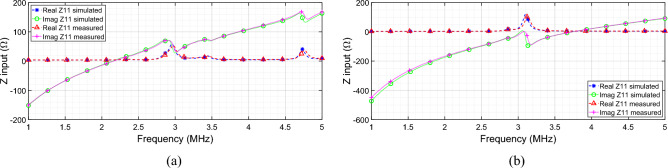
Comparison of real and imaginary components of the RF exciting coil input impedance: simulated and measured results for (**a**) the system that generates the focused distribution and (**b**) the system that produces the homogeneous field distribution.

As depicted in Fig. 10a,b, an excellent agreement was found for the driver input impedance between the numerical results and the experimental measurements, for both the “homogeneous” and the “focused” configurations.

**Figure 11:**
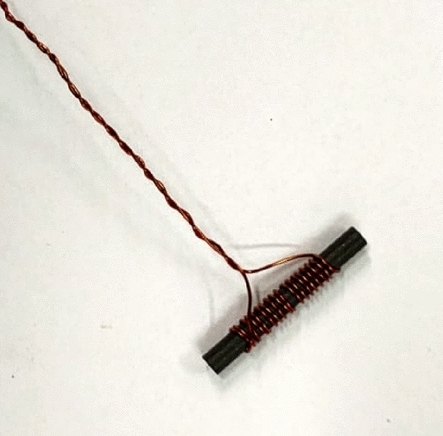
Fabricated (uncalibrated) magnetic field probe. A solenoid is wrapped around a tiny ferrite rod: the dimensions of the probe are much smaller than the unit-cell external diameter.

**Figure 12:**
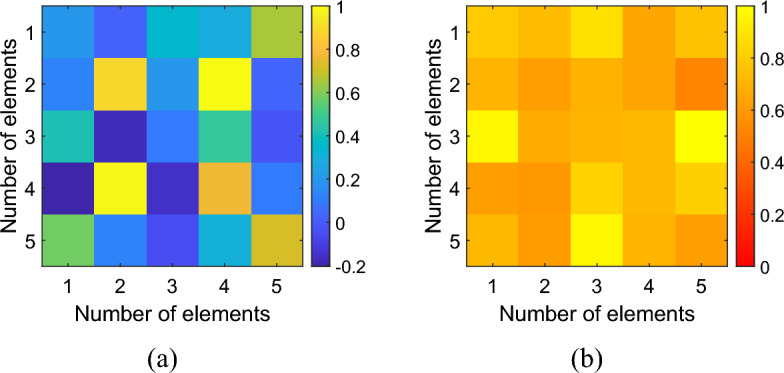
Measured normalized currents distribution within the metasurface unit-cells in the set-up which generates the focused magnetic field distribution (**a**) and the homogeneous field distribution (**b**).

In addition to the input impedance, an uncalibrated magnetic field probe was realized to examinate the current distribution in each cell of the metasurface; this aspect is essential to experimentally verify if the focusing and homogenization distributions are effectively accomplished. The magnetic field probe was designed based on the mutual impedance definition and Faraday’s law of induction. A tiny solenoid is wrapped around a small ferrite rod to create the sensor (Fig. 11). The probe’s dimensions (about 25 mm long and with a diameter of 5 mm) are sufficiently small with respect to the unit-cell external diameter; therefore, the probe does not significantly affect the field distribution. We measured the voltage drop at the end of the probing solenoid, when the probe is positioned above each unit-cell. As a matter of fact, the voltage drop *V*_*2*_ is proportional to the magnetic field generated by the single unit-cell that is linked by the solenoid area^52^:

5 $$Z_{21} = \frac{{V_{2} }}{{I_{1} }} = - \mu_{0} \frac{d}{dt}\oint {\vec{H} \cdot d_{n} /I_{1} }$$

where ${\mu }_{0}$ is the vacuum relative permeability, *H* (A/m) is the magnetic field intensity to be measured, ${d}_{n}$ is the differential surface element that is perpendicular to the loop, which is used to calculate the flux of the magnetic field through the unit-cell, ${I}_{1}$ is the current that produces the magnetic field and induces the voltage in the solenoid. Likewise, the unit-cell magnetic field ($\overrightarrow{H}$) is proportional to its circulating current (${I}_{1}$). Therefore, for both configurations, it has been possible to obtain the normalized unit-cell currents maps at the working frequency of 3 MHz by utilizing a VNA. The normalized distributions of the measured currents flowing within the cells of the “focused” and “homogeneous” configuration are depicted in Fig. 12. In general, a satisfactorily agreement has been observed with respect to simulations with a correlation greater than 80%. The slightly differences between full-wave and experimental currents distributions can be also attributed to the tolerances of the employed commercial capacitors (i.e., ± 1%).

**Figure 13:**
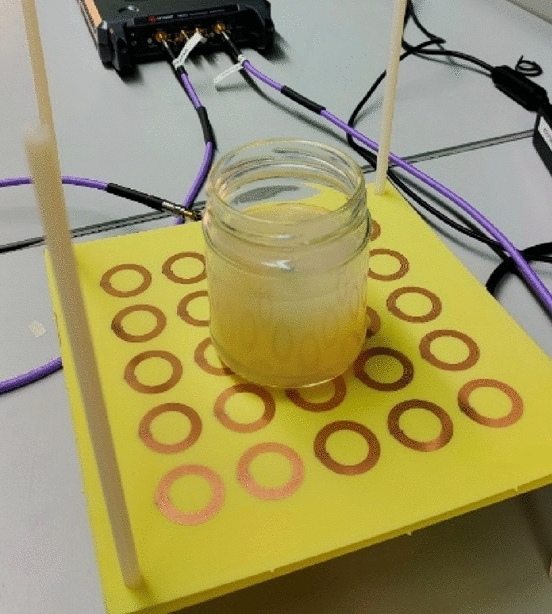
Fabricated phantom (MUT) to perform experimental measurements. The phantom has been realized by using de-ionized water and agar at 2% w/v.

Besides, to determine whether the proposed system (in its two configurations) can operate as a non-destructive sensing tool, additional measurements were performed on a phantom realized with de-ionized water and agar at a concentration of 2% w/v (Fig. 13). The phantom was created exploiting a cylindrical glass container measuring 6 cm in diameter and 8 cm in height.

Inside the MUT, a variable number of ferrite rods have been inserted to generate increasing inhomogeneities. The geometric specifications of the two distinct ferrite cylinders employed in the experimental set-up are reported in Table 3.

**Table 3:** Geometric size and permeability values of ferrite cylinders used as magnetic contrast for phantom experiments.

Parameters	Cylinder 1	Cylinder 2
Diameter (mm)	5	3
Length (mm)	20	25
µ	29	31

**Figure 14:**
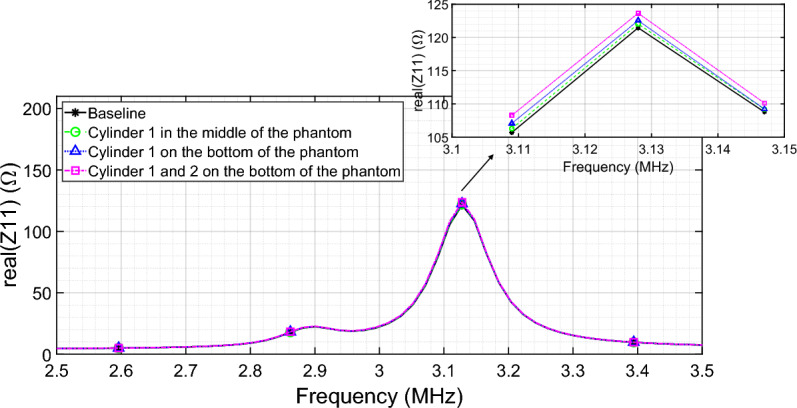
Variation of the amplitude of the RF coil input impedance real component with the homogenized metasurface as the number and position of the ferrite cylinders within the phantom change.

In particular, in order to assess the sensitivity of the system with the homogenized metasurface to the presence of the inclusion, an initial measurement was performed with the phantom without ferrite in it. The maximum resulting value of the real component of the measured input impedance ${Z}_{11}=121.5\Omega$ acted as a baseline to assess the system’s ability to detect the presence of the inclusion inside the MUT. Then, Cylinder #1 (Table 4) was placed in the center of the phantom (at 4 cm in height) and a measurement of the driver input impedance was acquired. After that, the same experiment was replicated with Cylinder #1 placed on the bottom of the phantom. Finally, in the third scenario, both Cylinder #1 and #2 were placed on the bottom of the MUT (as specified in the legend of the graph in Fig. 14). The sensing is performed by observing the variation in the amplitude of the exciting RF coil real part impedance peak value. For what concerns the homogenized metasurface, as shown in Fig. 14, the amplitude of the real part of the impedance increases as the amount of ferrite rods inside the phantom increases and as their positions are closer to the metasurface. The results are consistent with the physical principle underlying the system; moreover, it should be pointed out that the change in the system input impedance (from 122 to 123.6 Ω, which corresponds to a percentage impedance variation of 0.45% and 1.77% respectively, with respect to the baseline) is not too pronounced because we are working with a homogeneous filed distribution, which has a low sensitivity but a larger field of view. This means that we can observe a larger MUT with a single acquisition, but with a limited sensitivity.

**Table 4:** Percentage variation of the signal with respect to the reference baseline when the mut with a single ferrite inclusion is progressively moved over the focusing metasurface quadrants.

Quadrant	Variation (%)
1°	8
2°	2.4
3°	0.8
4°	0.6

**Figure 15:**
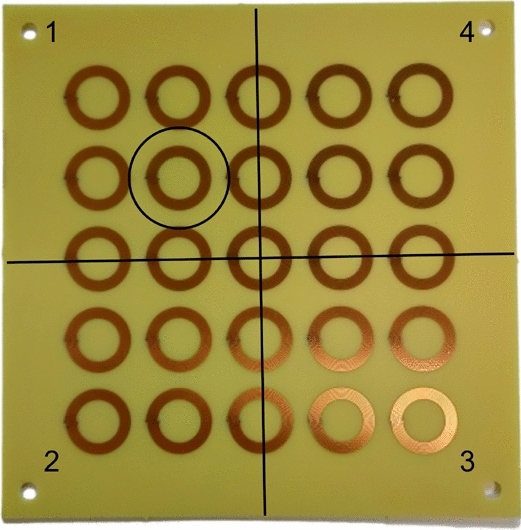
Metasurface configured to focus the magnetic field. The area in which the focal spot is produced has been circled, whereas the overall quadrants dividing the whole metasurface area are numbered from 1 to 4.

Next, we analyzed the configuration with the focusing metasurface. An initial measurement was performed with the phantom devoid of ferrites. As before, the maximum value of the driver input impedance (in real component) results equal to ${Z}_{11}=58.25\Omega$. This quantity was employed as the baseline for calculating the percentage variation of the system’s performance when Cylinder #1 (Table 3) is positioned inside the MUT. In this configuration, the metasurface area was divided into 4 quadrants (Fig. 15). The quadrant numbered 1 is relative at the position where the focal spot is present (see Fig. 2b for reference).

**Figure 16:**
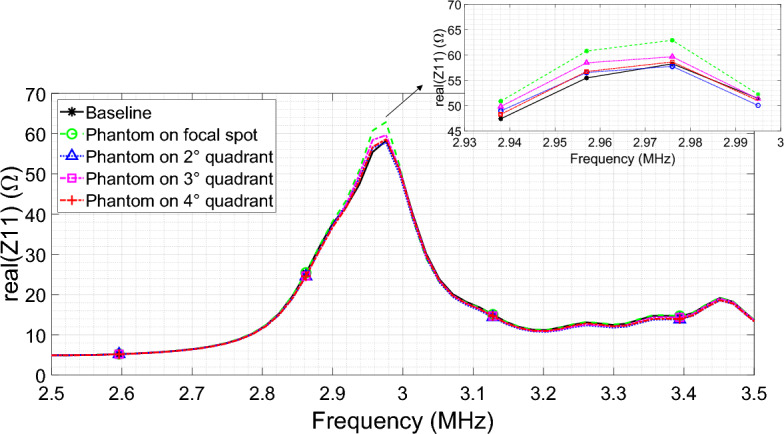
Real components of the RF coil input impedance comparison between the baseline (no ferrite) and phantom with ferrite progressively positioned on the four focusing metasurface quadrants.

Therefore, the measurements were conducted by positioning the phantom with one ferrite rod (Cylinder #1) in correspondence of each quadrant of the metasurface. The peak values of the real component of the driver input impedance at the metasurface resonance point (around 3 MHz) was recorded for each phantom position. Table 4 and Fig. 16 report the measured results, which indicate that the signal experiences the maximum percentage variation with respect to the baseline when the ferrite inclusion is in correspondence of the focal spot (+ 8%). Conversely, by placing the ferrite inclusion away from the focal spot quadrant, the percentage variation is progressively decreasing, with amplitude values of the real part of the RF exciting coil input impedance comparable to the baseline. Therefore, the focused metasurface has the capability to spatially localize the inhomogeneities, and it can be extremely useful in biomedical imaging as well as to scan a sample in industrial non-destructive testing evaluations. In particular, in a practical environment, it can be envisioned to make the metasurface focal spot dynamically reconfigurable with electronically controlled varactors, to perform a proper electronic scan of the MUT. Obviously, although the low frequency chosen for the design of the proposed system allows for a greater penetration depth within the MUT, this choice also brings some disadvantages when compared to systems that operate at higher frequencies, such as sensitivity and spatial resolution limitations. Moreover, since the primary goal of the study was to establish the technical feasibility of the sensing methodology, further research efforts are in program to assess practical implementation issues, as the ambient noise handling.

## Discussion

In this paper, we proposed a novel sensing technique based on low complexity and high-resolution passive holographic magnetic metasurface whose behavior can be reconfigured by modifying its response in the quasi-static regime. In particular, the magnetic field distribution produced by a closely placed and actively fed RF coil working at 3 MHz can be spatially filtered as desired for a specific application. The proposed methodology allows to find the reactive loads required to synthetize the target magnetic field distribution (for instance, focused or homogeneous) at the chosen geometrical plane.

The analytical framework, based on a magneto-static hypothesis and required to achieve the desired response control over the metasurface, was first described. Then, by performing accurate full-wave simulations, we demonstrated that it is possible to optimize the current distribution on the metasurface unit-cells in order to spatially control the magnetic field. We presented a test-case in which a 3 cm diameter focal spot can be created by opportunely loading the unit-cells with the proper capacitor. As a second example, the possibility of homogenizing the metasurface field response was also proved. These numerical results were supported by experimental validation performed on prototypes, fabricated by adopting standard PCB techniques and agar phantoms with ferrite cylinders as materials under test (MUT).

The proposed solution can guarantee a significant advantage in different sensing and/or diagnostic applications, both in industrial and biomedical environments, since it enables non-destructive detection of inhomogeneities in magnetically contrasted media. The system can be utilized in biomedicine as a theranostic instrument, to simultaneously diagnose and ablate different types of tumors. Another fascinating and rapidly emerging field of research where our solution can play an important role is the resonant inductive wireless power transfer. In this context, the present system can be implemented to increase the power transmission efficiency between transmitting and receiving coils. In addition, the ability to reconfigure the field distribution by exploiting the filtering capabilities of the metasurface may also allow the recharging of a single device while isolating additional electronics in the nearby. Future research will also investigate the possibility of realizing metasurfaces having real-time reconfigurability by loading the unit-cells with variable lumped elements, e.g., varactor diodes. The ability to dynamically focus (or, on the contrary, homogenize) the beam at any point within the near-field region of the metasurface would make the proposed system suitable for a number of emerging applications, such as microwave absorbers, able to protect sensitive antennas or electronics from undesired interferences. Moreover, electronically varying the magnetic field distribution would be advantageous in terms of scanning times reduction and increased system robustness. Finally, also purely dielectric inhomogeneities detection will be also evaluated by appropriately optimizing the metasurface design. As concluding remarks, it is fundamental to focus future efforts towards addressing practical challenges that emerge from real-world applications. In this regard, significant attention will be given to examining the specific sources of environmental noise and enhancing the stability of the proposed system against such deviations.

## Data Availability

The datasets used and/or analyzed during the current study are available from the corresponding author on reasonable request.
